# Stabilization of Olive Oil in Water Emulsion with Dairy Ingredients by Pulsed and Continuous High Intensity Ultrasound

**DOI:** 10.1021/acsomega.3c00227

**Published:** 2023-03-14

**Authors:** Ummu Busra Yavuz, Erenay Erem, Meral Kilic-Akyilmaz

**Affiliations:** Department of Food Engineering, Istanbul Technical University, 34469 Istanbul, Türkiye

## Abstract

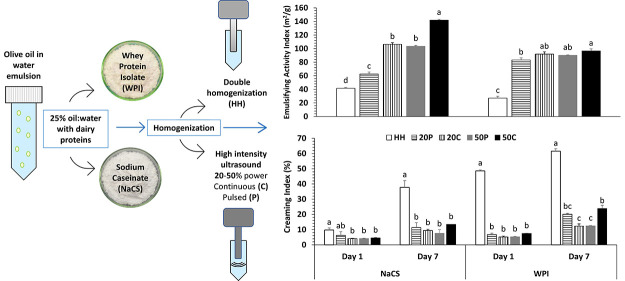

Application of high intensity ultrasound (HIUS) for stabilization of olive oil in water emulsion with different dairy ingredients including sodium caseinate (NaCS) and whey protein isolate (WPI) was investigated. The emulsions were prepared by homogenization with a probe and then treated with either a second homogenization or HIUS at a different power level (20 and 50%) in pulsed or continuous mode for 2 min. The emulsion activity index (EAI), creaming index (CI), specific surface area (SSA), rheological properties, and droplet size of the samples were determined. The temperature of the sample rose when HIUS was applied in continuous mode and at increasing power level. HIUS treatment increased EAI and SSA of the emulsion and decreased droplet size and CI compared with those of the double-homogenized sample. Among the HIUS treatments, the highest EAI was found in the emulsion with NaCS that was treated at a power level of 50% in continuous mode, and the lowest one was obtained by HIUS applied at a power level of 20% in pulsed mode. SSA, droplet size, and span of the emulsion were not affected by HIUS parameters. Rheological properties of HIUS-treated emulsions were not different from those of the double-homogenized control sample. Continuous HIUS at 20% power level and pulsed HIUS at 50% power level reduced creaming in the emulsion after storage at a similar level. HIUS at a low power level or in pulsed mode can be preferred for heat sensitive materials.

## 1 Introduction

The oil-in-water (O/W) emulsions which consist of a dispersion of oil in an aqueous phase are found commonly in food products such as beverages, mayonnaise, salad dressing, and margarine. Emulsion-based food products are thermodynamically unstable, which leads to phase separation over time.^1^ Therefore, they are generally stabilized by the addition of emulsifiers. Sodium caseinate (NaCS) is a commonly used dairy-based emulsifier. It is produced by acidification of casein and then neutralization with sodium hydroxide.^2^ NaCS stabilizes emulsions by adsorbing at the O/W interface and producing thick interfacial films by hydrophobic interactions and calcium phosphate nanocluster bridges.^3,4^ Whey protein isolate (WPI) produced from whey, a byproduct from the dairy industry, is widely preferred as a natural emulsifier in the food industry due to its high capacity of adsorption onto the oil–water interface.^5^ WPI can form a viscoelastic interfacial film on the surface of oil droplets by hydrogen bonds and hydrophobic and electrostatic interactions.^6^ Although dairy-based emulsifiers are used in food formulations, there is a need for high energy emulsification methods to form a stable emulsion due to their slow reaction into the interface.^7^ High intensity ultrasound (HIUS) has been applied for stabilization of emulsion systems with dairy-based emulsifiers.^8,9^

HIUS is a nonthermal food processing technique that applies acoustic energy waves at low frequency (16–100 kHz) and high power intensity (10–1000 W/cm^3^) created by piezoelectric transducers. Cavitation is the major mechanism of action of HIUS where passage of sound waves through a medium creates and grows bubbles which collapse violently. As a result, a local increase in temperature and pressure, shockwaves, turbulence, and shear forces occur upon bubble collapse.^10^ In addition, sound waves passing through a medium also create noncavitational effects including mechanical vibration and acoustic streaming. These physical forces can disperse protein particles, increase solubility, and improve emulsifying properties. HIUS treatment can also unfold proteins and expose sulfhydryl groups, which lead to the production of a strong protein layer at the O/W interface and improvement of the emulsion stability.^11^ HIUS can be applied in two distinct modes which are continuous and pulsed. In the pulsed mode, the ultrasound processor is turned on and off intermittently during the process, which results in reduced heat absorption by the sample.^12^ Moreover, pulsed mode is reported to be more advantageous than continuous mode, which can improve efficiency and lower energy consumption in some cases, and it causes fewer technical issues such as depreciation of the equipment and erosion of the horn tip.^13^

Although positive effects of HIUS on food emulsions have been shown, studies about effects of process parameters, especially mode of operation, on the stability of emulsions are scarce. Recoalescence and disruption of droplets take place cocurrently during the process of emulsification, and final droplet size is affected by the kinetics of each event.^14^ For preventing overprocessing that can cause instability, optimum energy input needs to be decided.^14^ When the power level or duration of HIUS is increased for intensifying its effect on a material, part of the energy is absorbed as heat by the material. If HIUS is applied in pulsed mode with less heat absorption by the material, the sole effect of acoustic waves can be obtained, heat-sensitive materials can be protected, and energy consumption and cost of the process can be reduced. This study aims to determine the effects of power level (20 or 50%) and operation mode (pulsed or continuous) of HIUS on the formation and stability of olive oil in water emulsions with different dairy ingredients, NaCS and WPI.

## 2 Materials and Methods

### 2.1 Materials

Extra virgin olive oil with an acidity less than 0.8% was obtained from a local retail store (Istanbul, Türkiye). WPI (Prolacta 95LL Instant, Lactalis Ingredient, Bourgbarré, France) and NaCS (Maysa Gıda, Istanbul, Türkiye) were used as emulsifying proteins. WPI contained 93.0% protein, 4.4% moisture, 1.7% ash, 0.4% fat, and 0.5% lactose. NaCS had 90.5% protein, 6.0% moisture, 1.5% ash, 1.5% fat, and 0.5% lactose. All of the chemicals used were of analytical grade. Deionized water was used for the preparation of all solutions.

### 2.2 Preparation of Emulsion

Solutions of NaCS and WPI (3%, w/v) were prepared by dissolving them in a phosphate buffer. Sodium dihydrogen phosphate dihydrate and disodium hydrogen orthophosphate were used to prepare a phosphate buffer solution (pH 7.0, 0.1 M). Protein solutions were kept at 4 °C overnight to ensure complete hydration.

Oil in water emulsions were prepared using 3 mL of olive oil and 9 mL of protein solution (25% (v/v) oil volume fraction) in 50 mL tubes in two stages. In the first stage, homogenization was applied with a probe-homogenizer at 15500 rpm for 2 min (Ultraturrax T18, IKA Werke, Staufen, Germany). In the second stage, a second homogenization (control) or HIUS was applied at 20 or 50% amplitude levels in pulsed (0.5 s on/0.5 s off) or continuous mode for 2 min. The HIUS system consisted of a generator unit operating at a maximum power of 400 W and a frequency of 24 kHz (UP400S Hielscher Ultrasonics GmbH, Germany) and a 14-mm-diameter cylindrical titanium sonotrode (H14, maximum amplitude 125 μm, maximum acoustic power density 105 W/cm^2^, Hielscher GmbH, Germany). The probe was immersed in the center of the tube over the oil–water interface approximately at a depth of 1 cm from the surface. Samples were placed in an ice–water bath to prevent overheating during HIUS treatment. A thermocouple was used for measuring the temperature of the samples immediately after treatments. Acoustic power intensity transmitted to the sample was measured by the calorimetric method at room temperature by using distilled water.^15^ HIUS parameters applied in the experimental trials are shown in Table 1.

**Table 1:** Samples and Average Temperature after Treatment by HIUS at Room Temperature for 2 min

power level (%)	mode	sample code	temperature (°C)	power intensity (W/cm^2^)
		HH (control)^a^	23	
20	pulsed	20P	23	13.6
20	continuous	20C	43	27.2
50	pulsed	50P	37	22.6
50	continuous	50C	50	54.3

a HH: Double homogenization.

### 23 Emulsion Activity Index

The emulsion activity index (EAI) was measured at room temperature by using the method of Pearce and Kinsella.^16^ An aliquot of 50 μL of emulsion was taken from the bottom of the tube and diluted with 20 mL of 0.1% (w/v) sodium dodecyl sulfate (SDS) solution. Further dilutions were made if required to keep the absorbance value under 0.4. Absorbance of diluted samples was measured immediately by using a spectrophotometer (Biospec 1601 UV–vis Spectrophotometer, Shimadzu, Kyoto, Japan) at 500 nm. Turbidity was calculated by following eq 1:
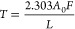
1where *T* is turbidity, *A*_0_ is the absorbance of diluted emulsion at 500 nm, *F* is the dilution factor, and *L* is the path length of the cuvette (m).

EAI (m^2^/g) was calculated according to Sui et al.^17^ by following eq 2:
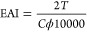
2where ϕ is the volume fraction of oil in the emulsion and *C* is the concentration of the protein (g/mL).

### 2.4 Creaming Index

Fresh emulsion samples (12 mL) were stored in glass tubes (15 mm diameter, 50 mm length) at 4 °C. Measurements were taken with a turbidimeter (TurbiScan Classic MA 2000, Formulaction, l’Union, France) at 24 h and 7 days. Separated serum layer height at the bottom (*H*_S_) and total emulsion height (*H*_T_) were determined by the change in light backscattering value. The creaming index (CI) was calculated according to Keowmaneechai and McClements^18^ by following eq 3.
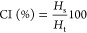
3where *H*_s_ is the bottom serum layer height and *H*_t_ is the total emulsion height.

### 2.5 Rheological Properties

Rheological properties of freshly prepared emulsions (without phase separation) were measured with a rheometer (Haake RheoStress 1, Karlsruhe, Germany) at 23 °C using a cone and plate sensor (C35/2, 35 mm diameter, 2°, Thermo Fisher Scientific, Karlsruhe, Germany). The flow curve of the emulsions was obtained by increasing the shear rate from 0.1 to 300 s^–1^ linearly. The obtained data from the flow curve was fitted to the power law, eq 4:
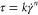
4where τ is the shear stress (Pa), *k* is the consistency index (Pa s^*n*^), γ̇ is the shear rate (s^–1^), and *n* is the flow behavior index.

### 2.6 Droplet Size

Oil droplet size in the emulsions was measured immediately after preparation. Emulsions were diluted with SDS solution (0.1%, w/v) at a 1:2 ratio before measurement. Measurements were done by using a light microscope with a magnification of 100× (Nicon Ni–U, Nikon Instruments, Amsterdam, The Netherlands). Images from diluted emulsions at six different zones were captured by using a connected camera (Nicon DS-U3, Nikon Instruments Europe B.V., Amsterdam, The Netherlands). The diameters of approximately 1000 oil droplets in each image were measured with the software of the microscope. Data from number-weighted distribution were converted to volume-weighted distribution. Frequency and cumulative distribution curves were obtained from the data.

The mean droplet diameters were calculated as *D*_4,3_ (volume mean diameter) and *D*_3,2_ (surface mean diameter) by following eqs 5 and 6, where *n*_*i*_ is the number of droplets that have the same geometric diameter (*d*_*i*_):
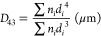
5
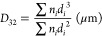
6

Specific surface area (SSA) of the droplets was calculated according to the following equation and expressed as square meters per milliliter of emulsion (eq 7):^19^
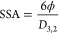
7where ϕ is the oil volume fraction of the emulsion.

Variation in distribution of the droplet size, span, was calculated according to eq 8.
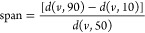
8where *d*(*v*,10), *d*(*v*,50), and *d*(*v*,90) are diameters at 10%, 50%, and 90% cumulative volume, respectively.

### 2.7 Statistical Analysis

Experimental trials were carried out in triplicate, and measurements were repeated at least two times. Effects of treatment parameters on measured properties of the samples were analyzed by Anova (IBM SPSS Statistics 24, New York, USA). Means were compared according to Tukey’s test. A significance level of 0.05 was used in the analysis.

## 3 Results and Discussion

### 3.1 Temperature Change during HIUS

HIUS resulted in a temperature increase in the range of 37–50 °C depending on the process parameters (Table 1). HIUS applied at a 20% power level in pulsed mode did not cause a change in temperature after the treatment, similar to double homogenization. On the other hand, when the power was increased from 20 to 50%, an increase in temperature to 37 °C was also observed in pulsed mode, but to a lower extent than that by continuous mode (50 °C). Absorption of part of the sound energy by the emulsion medium caused heating and an increase in the temperature of the samples. An excessive temperature increase can influence the quality and stability of an emulsion and its heat-sensitive components.^7^

### 3.2 Droplet Size

Both volume mean diameter (*D*_4,3_) and surface mean diameter (*D*_3,2_) were measured. *D*_4,3_ is more sensitive to the presence of large particles, while *D*_3,2_ is related to the interfacial area of the emulsion. Both mean diameters of oil droplets decreased significantly by application of HIUS compared with those of the double-homogenized control sample (Table 2). Similar results were reported by McCarthy et al.^20^ and Sun et al.^21^ HIUS is noticeably an effective technology in the formation of stable emulsions resistant to coalescence by fast absorption of protein at the interface that lowers interfacial tension and droplet size.^22^

**Table 2:** Span, Mean Droplet Size, and Specific Surface Area (SSA) of Olive Oil in Water Emulsions Containing Na-Caseinate (NaCS) and Whey Protein Isolate (WPI)^a^

sample	span	*D*_4,3_ (μm)	*D*_3,2_ (μm)	SSA (m^2^/mL)
NaCS-HH	1.08 ± 0.18^a^	10.72 ± 0.45^a^	8.69 ± 0.72^a^	0.17 ± 0.01^a^
NaCS-20C	0.95 ± 0.20^a^	3.55 ± 0.19^b^	3.09 ± 0.31^b^	0.48 ± 0.01^b^
NaCS-50C	0.69 ± 0.13^a^	3.77 ± 0.19^b^	3.40 ± 0.14^b^	0.48 ± 0.03^b^
NaCS-50P	0.72 ± 0.14^a^	3.47 ± 0.18^b^	3.11 ± 0.17^b^	0.44 ± 0.01^b^
WPI-HH	1.20 ± 0.06^a^	8.76 ± 0.30^a^	7.01 ± 0.20^a^	0.21 ± 0.01^a^
WPI-20C	1.30 ± 0.15^a^	2.72 ± 0.55^b^	2.19 ± 0.23^c^	0.69 ± 0.06^b^
WPI-50C	0.93 ± 0.06^a^	3.00 ± 0.18^b^	2.64 ± 0.16^b^	0.72 ± 0.05^b^
WPI-50P	1.14 ± 0.09^a^	2.49 ± 0.22^b^	2.08 ± 0.11^c^	0.57 ± 0.03^b^

a HH, double homogenization; 20C, 20% power continuous; 50C, 50% power continuous; 50P, 50% power pulsed. Means marked with different letters are different for each ingredient (*P* < 0.05).

There was no significant difference between oil droplet sizes of emulsions treated with HIUS at different power level or operation mode (*p* > 0.05), with the exception of the emulsion sample with WPI treated by continuous HIUS at a 50% power level. This sample had a slightly larger mean droplet size (*D*_3,2_) compared to the other samples with WPI. Similarly, Sui et al.^17^ reported an increase in particle size by increasing HIUS power from 300 to 450 W for 24 min for an oil in water emulsion stabilized with soy protein isolate and lecithin. On the other hand, Kaltsa et al.^14^ reported that *D*_3,2_ values of an emulsion were reduced by increasing the energy density from 40 to 100% of HIUS. They explained this not only by intense cavitation but also elevation in temperature decreasing viscosity, interfacial tension, and Laplace pressure. Xiong et al.^23^ demonstrated that, while HIUS application at 20 kHz for 12 min decreased the size of droplets in an emulsion, when the power was increased from 150 to 300 or 600 W, it increased the size of the droplets. Extensive duration or temperature increase by HIUS can cause coalescence of droplets due to partial protein denaturation and their flocculation as in the sample with WPI treated by continuous HIUS at the 50% power level.^20^ However, overall findings in this study differ from those of reported studies possibly due to the lower power and duration applied.^24^

**Figure 1:**
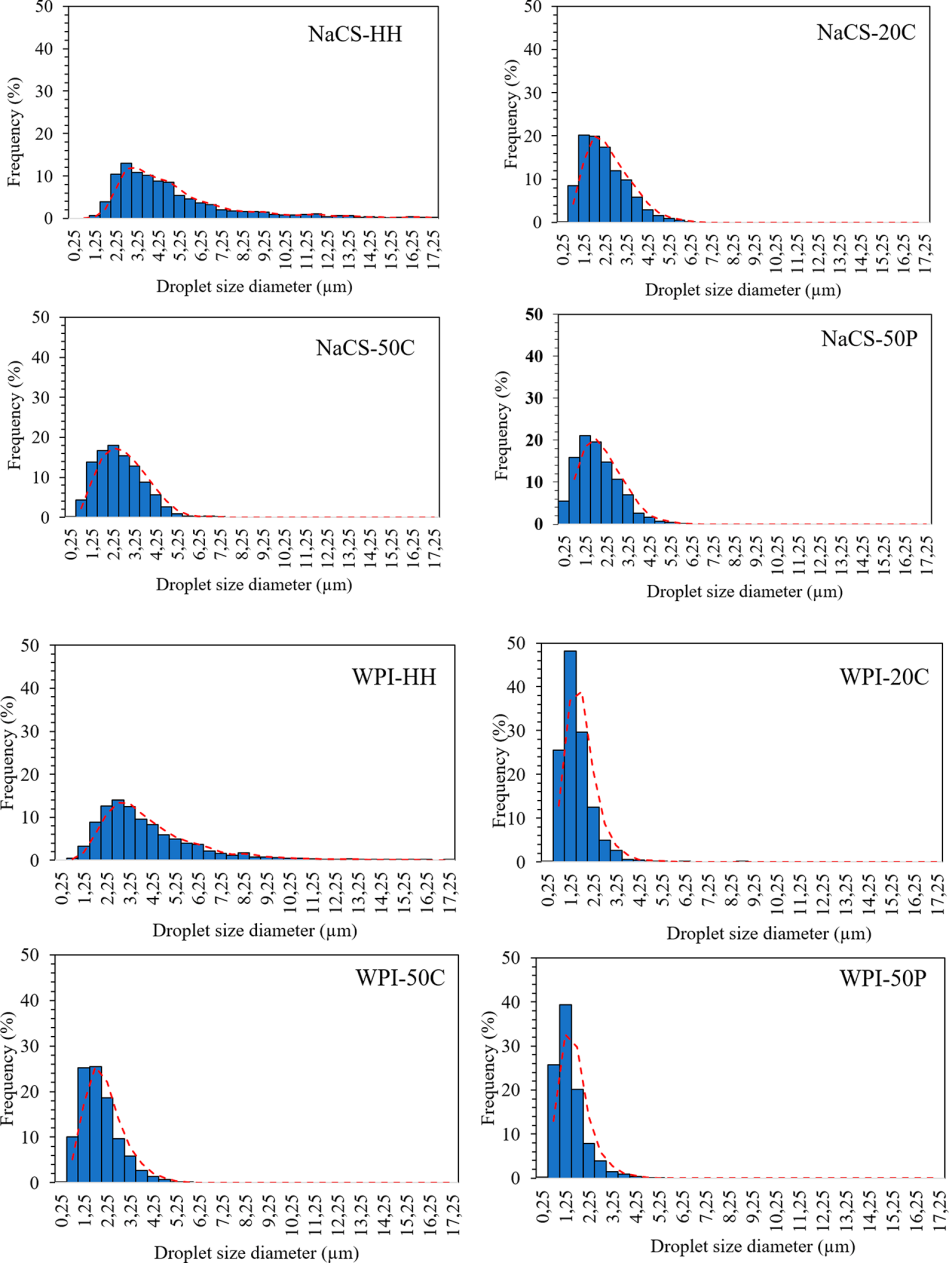
Droplet size distribution of NaCS and WPI containing oil in water emulsions (HH, double homogenization; 20C, 20% power continuous; 50C, 50% power continuous; 50P, 50% power pulsed).

HIUS treatments resulted in narrower droplet size distribution in the emulsion regardless of protein ingredient (Figure 1). Droplet size of the emulsion with WPI was smaller compared to that with NaCS. Whey proteins are water-soluble, small-sized proteins compared to caseins, which have a micellar structure. Similar results were reported by Aslan and Dogan^24^ and Li et al.^8^ HIUS was found to break down aggregated milk protein particles and reduce particle size.^10,25,26^

Span is a similar measurement to the polydispersity index (PDI), representing the size distribution of an emulsion. The value is close to zero when the particles in an emulsion are homogeneous and monodisperse. Polydisperse systems have higher span values and a greater tendency to aggregation than monodisperse systems. For both ingredients, a monomodel distribution was observed, and there was no difference between span values of the control and the ultrasound-treated samples (*p* > 0.05). NaCS resulted in lower span values, which indicates a more homogeneous emulsion with similar droplet size compared to WPI. Hennemann et al.^27^ reported that when the power level of HIUS increased from 20 to 40%, the span value increased slightly due to high energy, leading to more collision and aggregation of particles. The span was also close to 1 in this study, which shows heterogeneity of the droplet size, especially in the case of WPI. Increased particle interactions induced by disruption of particles by HIUS might cause a wide particle size distribution, although mean particle size was reduced.

Specific surface area (SSA) represents the total surface area of the droplets in an emulsion and knowledge about the amount of required emulsifier for covering the surface.^28^ A reduction in droplet diameter (*D*_3,2_) causes an increase in SSA and stability of an emulsion.^29^ SSA of the emulsion increased with HIUS application for both protein ingredients compared with that of the control sample treated with double homogenization. HIUS effectively increased SSA by reducing the droplet size regardless of power level and mode of operation applied.^30^

### 3.3 Emulsion Activity Index

**Figure 2:**
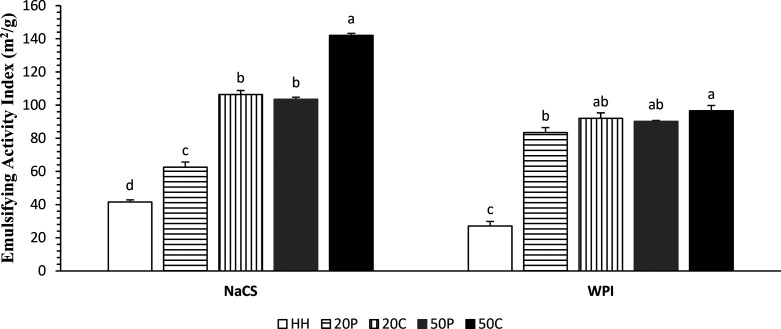
Emulsion activity index values of emulsions containing different protein ingredients treated with HIUS (HH, double homogenization; 20C, 20% power continuous; 50P, 50% power pulsed; 50C, 50% power continuous). Means marked with different letters are different for each protein ingredient (*P* < 0.05).

EAI gives information about the absorption of a protein at the O/W interface and represents interfacial area stabilized by per unit mass of a protein. EAI values of all HIUS-treated emulsions were higher than that of the control sample for each protein ingredient (Figure 2). HIUS treatment at the power level of 50% in continuous mode provided the highest EAI for both NaCS and WPI, followed by 20% continuous, 50% pulsed, and 20% pulsed treatments. Similar results were reported for O/W emulsion with whey protein concentrate by Mekala et al.,^31^ with soy protein isolate by Sui et al.,^17^ and with NaCS by Furtado et al.^32^ A larger impact of HIUS conditions were observed in the case of NaCS compared to WPI, which could be related to more dissociation of large casein aggregates by HIUS.

### 3.4 Creaming Index

**Figure 3:**
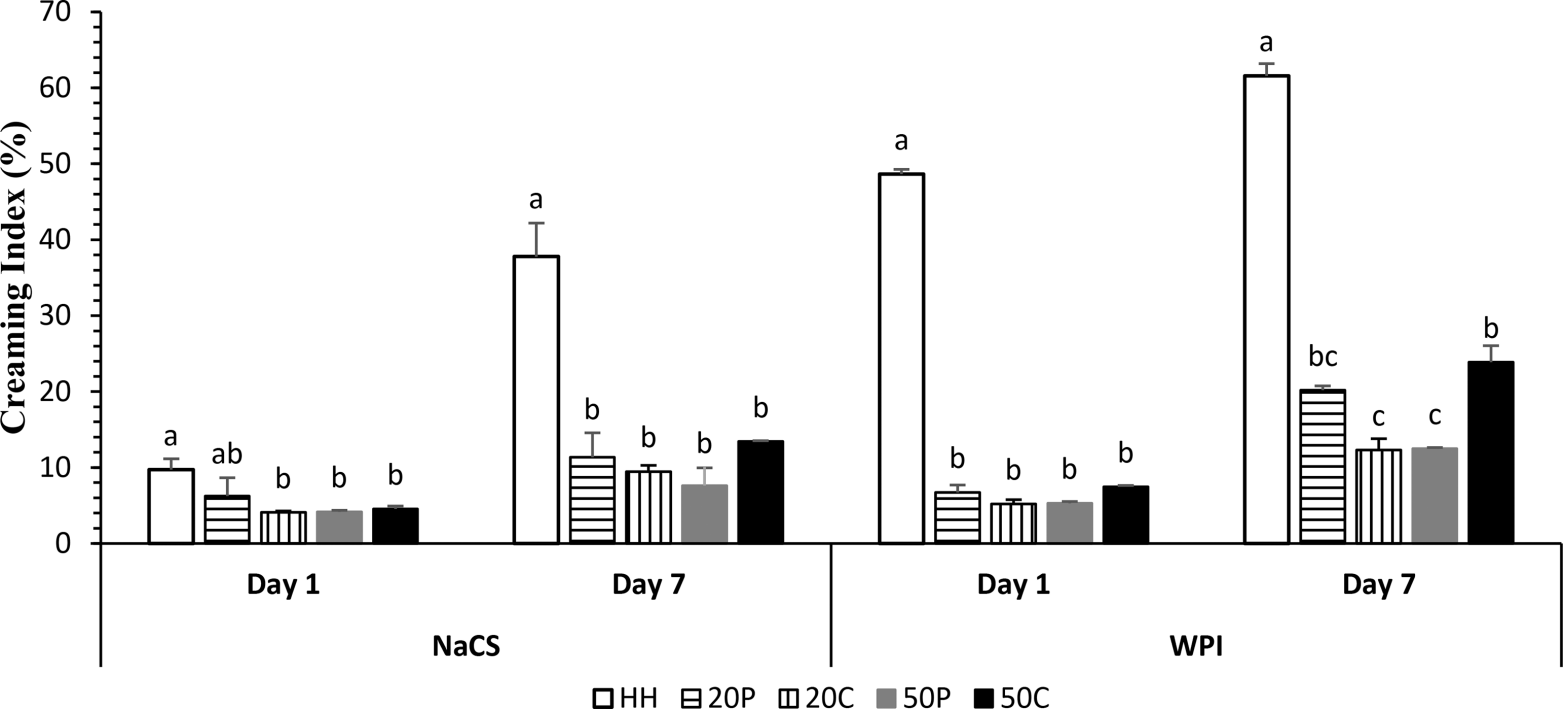
Creaming index values of emulsions containing different protein ingredients treated with HIUS at days 1 and 7 (HH, double homogenization; 20C, 20% power continuous; 50C, 50% power continuous; 50P, 50% power pulsed). Means marked with different letters are different for each protein ingredient (*P* < 0.05).

Flocculation and coalescence can occur spontaneously in an emulsions, causing creaming during the storage period. The CI value gives indirect information about droplet aggregation. Aggregation of droplets accelerates the occurrence and increases the height of the cream phase. CI significantly decreased by HIUS treatments for both protein ingredients compared to double homogenization (Figure 3, *p* < 0.05). Fu et al.^33^ and Chen et al.^34^ reported similar findings. A large increase in CI was observed after 7 days of storage in the control sample, while lesser increases were observed in the HIUS treated samples. HIUS was found to be capable of preventing creaming, which could be explained by the reduction in droplet size and possible increase in repulsive forces between droplets.^17^

The emulsion with NaCS treated with pulsed HIUS at 20% power level had a CI value similar to that of the control sample at day 1; however, the control sample exhibited a greater increase after storage. On the other hand, continuous HIUS at the 50% power level increased CI in the emulsions with both ingredients. Disrupted proteins by intense HIUS treatment was possibly aggregated during storage and gave rise to creaming in the emulsion. On the contrary, Cabrera-Trujillo et al.^35^ found that continuous application of HIUS resulted in the lowest phase separation in an emulsifier-free O/W emulsion followed by 30 s/30 s and 20 s/20 s pulsed treatments. However, they did not have an emulsifier ingredient and stored the samples for only 3 h. The effect of HIUS on proteins should be considered for emulsions with dairy proteins as in the current study.

NaCS yielded lower CI compared to WPI. Thaiwong and Thaiudom^36^ also reported that NaCS enabled the most stable emulsions compared to whey proteins. This was explained by the structural size of NaCS, which can move to the interfacial surface and stabilize the emulsion. In emulsions with WPI, there was no difference between CI values of HIUS-treated samples, but after storage, HIUS-treated samples at the power level of 50% in continuous mode and at 20% in pulsed mode yielded similar CI values. Interestingly, the samples treated with HIUS at 20% in continuous mode and 50% in pulsed mode had the lowest CI values. These results indicated that treatment of the emulsion with continuous HIUS at a 50% power level caused instability, possibly by affecting the structure of the protein. Whey proteins are heat-labile and denature at temperatures above 60 °C. Even though the average temperature measured did not exceed 50 °C, local temperature increases can affect the structure of whey proteins.

### 3.5 Rheological Properties

**Figure 4:**
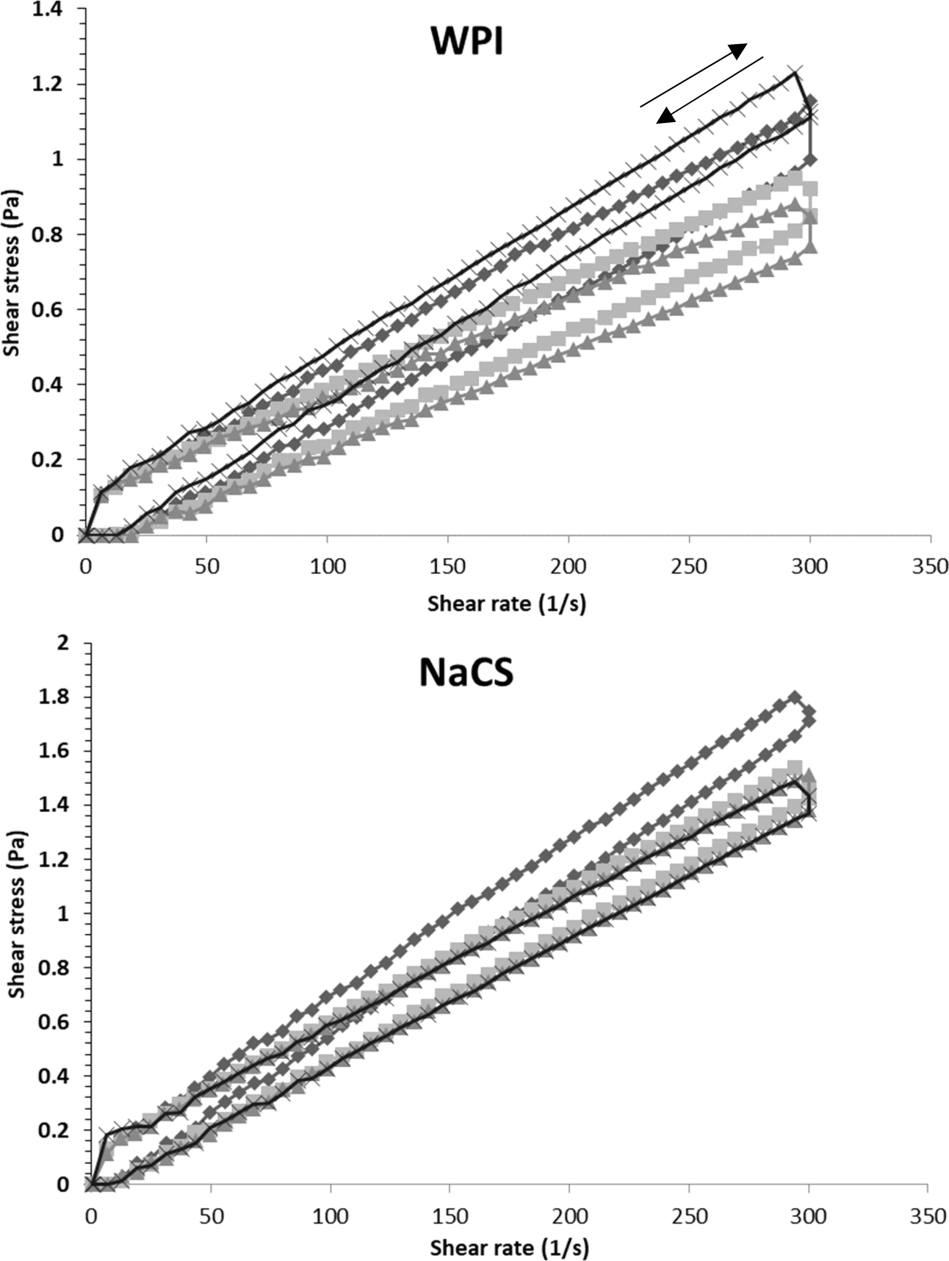
Flow curves of emulsions containing NaCS and WPI treated with HIUS. (⧫) HH, double homogenization; (■) 20C, 20% power continuous; (▲) 50P, 50% power pulsed; (×) 50C, 50% power continuous.

The shapes of the flow curves of the control and HIUS treated emulsions were similar even though shear stress values of HIUS-treated emulsions were found to be slightly lower than those of the control sample (Figure 4). HIUS treatment did not cause a significant change in rheological behavior of the emulsion samples. However, the emulsion with WPI treated with continuous HIUS at 50% power showed higher shear stress values than the other samples. This could be related with changes in protein structure due to relatively more intense ultrasound treatment. Acoustic cavitation can cause fast molecular movement and unfolding of protein chains that leads to an increase in viscosity.^17^

Consistency coefficient, flow behavior index, and thixotropy of the control and HIUS-treated samples were similar (Table 3). All emulsions exhibited pseudoplastic behavior with a flow behavior index below 1. This indicates that the emulsions are sensitive to shear forces, which can result in reduced stability. Similar findings were reported by Xiong et al.^23^ and Aslan and Dogan.^24^ Furtado et al.^32^ also did not find a significant change in consistency by increasing intensity of ultrasound. However, Aslan and Dogan^24^ observed that HIUS treatment of an emulsion prepared with olive oil in a dairy-based ingredient reduced the consistency coefficient. Similarly, Kumar et al.^37^ and Kaltsa et al.^14^ reported that viscosity was decreased after HIUS treatments in mayonnaise emulsions with xanthan and guar gum, which was explained by the increase in temperature. In this study, there was no excessive temperature increase or a hydrocolloid stabilizer, which could effect the structure of the emulsion.

**Table 3:** Rheological Properties of Olive Oil in Water Emulsions Containing Na-Caseinate (NaCS) and Whey Protein Isolate (WPI)^a^

sample	consistency index, *k* (mPa s)	flow behavior index, *n*
NaCS-HH	14.00 ± 0.41^a^	0.856 ± 0.001^a^
NaCS-20C	13.31 ± 0.61^a^	0.831 ± 0.007^a^
NaCS-50P	13.34 ± 0.91^a^	0.829 ± 0.010^a^
NaCS-50C	13.99 ± 0.62^a^	0.820 ± 0.004^a^
WPI-HH	11.31 ± 1.22^a^	0.792 ± 0.039^a^
WPI-20C	12.13 ± 0.71^a^	0.781 ± 0.007^a^
WPI-50P	11.35 ± 0.22^a^	0.767 ± 0.013^a^
WPI-50C	12.38 ± 1.34^a^	0.788 ± 0.024^a^

a HH, double homogenization; 20C, 20% power continuous; 50C, 50% power continuous; 50P, 50% power pulsed. Means marked with different letters are different for each ingredient (*P* < 0.05).

## 4 Conclusions

HIUS treatment stabilized the emulsion of olive oil in water with NaCS or WPI compared to conventional probe homogenization. HIUS improved the emulsifying activity of the proteins and reduced creaming in the emulsion by decreasing oil droplet size, thus increasing the specific surface area of the interface. Different power levels and application modes of HIUS treatment did not affect droplet size, span, SSA, or rheological properties of the emulsion. Continuous HIUS at a power level of 50% yielded the highest emulsifying activity; however, it also induced more creaming after storage for 7 days. Pulsed HIUS at a 50% power level and continuous HIUS at a 20% power level were found equivalent in terms of their effect on emulsifying activity index and creaming index. Pulsed mode can be preferred for emulsion formation to prevent an excessive increase in temperature, which can be detrimental to protein structure and emulsion stability. Optimization of HIUS parameters is recommended not only for achieving the highest stability but also reducing energy consumption in production of an emulsion. Findings of the study can be of use for emulsion-based food, chemical, nutraceutical, or pharmaceutical products. Moreover, use of direct continuous flow or indirect treatment systems can be investigated to reduce the effect of HIUS on heat-sensitive components.
